# Supplementary material for: Effect of Strobilanthes tonkinensis Lindau Addition on Black Tea Flavor Quality and Volatile Metabolite Content
Source: Foods. 2022 Jun 7;11(12):1678. doi: 10.3390/foods11121678 (PMC9222377; doi:10.3390/foods11121678)
Supplement: Supplementary file 1 [file foods-11-01678-s001.zip › foods-1718320-supplementary.pdf]

Table S1. List of the all the volatile metabolites in BT, BoS, and STL.

No.	Name	Retention Time (min)	RI[A]	RI[B]	CAS number	BT	BoS	STL
1	Dimethyl ether	2.15	699	-	115-10-6	0.011±0.005b	0.023±0.022a	0.022±0.011a
2	3-methyl-butanal	2.20	700	678	590-86-3	0.256±0.042b	0.296±0.050a	0.057±0.006c
3	2-methyl-butanal	2.28	702	682	96-17-3	0.305±0.027b	0.351±0.210a	0.081±0.011c
4	Anhydride propanoic acid	2.41	706	-	123-62-6	0.183±0.016a	0.171±0.085a	0.040±0.004b
5	Pentanal	2.57	711	701	110-62-3	0.051±0.006a	0.041±0.020a	0.021±0.003b
6	Furan, 2-ethyl-	2.62	712	712	3208-16-0	0.057±0.003a	0.052±0.003a	0.006±0.001b
7	1-Butanol, 3-methyl-	3.02	763	734	123-51-3	0.020±0.002a	0.018±0.010a	0.002±0.002b
8	1-Butanol, 2-methyl-	3.07	765	762	137-32-6	0.005±0.001a	0.005±0.003a	0.006±0.009a
9	1-Pentanol	3.56	778	768	71-41-0	0.040±0.004a	0.035±0.014a	0.016±0.002b
10	Toluene	3.61	780	773	108-88-3	0.076±0.022b	0.089±0.011a	0.051±0.008c
11	4-Hexen-3-one	3.66	781	-	2497-21-4	0.015±0.002a	0.013±0.004a	0.003±0.000b
12	Furan, 2-methoxy-	4.28	799	-	25414-22-6	0.028±0.001b	0.045±0.014a	0.004±0.000c
13	Hexanal	4.30	799	800	66-25-1	0.396±0.020a	0.223±0.084b	0.042±0.007c
14	(E)-2-hexenal	5.96	846	854	6728-26-3	1.214±0.039a	0.661±0.117b	0.042±0.008c
15	1-Methyl-1H-1,2,4-triazole	6.07	849	-	6086-21-1	1.214±0.039a	0.661±0.117b	0.042±0.008c
16	(E)-3-hexen-1-ol	6.08	849	844	928-97-2	0.774±0.062b	0.963±0.229a	0.058±0.011c
17	Ethylbenzene	6.25	854	857	100-41-4	0.004±0.000a	0.003±0.001a	0.003±0.001a
18	cis-2-methyl- cyclopentanol	6.44	860	-	25144-05-2	0.085±0.006b	0.176±0.036a	0.011±0.015a
19	1-hexanol	6.52	862	879	111-27-3	0.489±0.019b	0.751±0.191a	0.020±0.005c
20	Styrene	7.32	884	890	100-42-5	0.020±0.001b	0.015±0.001b	0.036±0.003a
21	p-Xylene	7.40	887	888	106-42-3	0.006±0.000a	0.004±0.001a	0.001±0.002b
22	Heptanal	7.76	897	903	111-71-7	0.093±0.003a	0.068±0.016b	0.008±0.001c
23	Methoxy-phenyl-oxime_	7.86	900	-	1000222-86-6	0.105±0.030a	0.078±0.008b	0.021±0.011c
24	Pyrolo[3,2-d]pyrimidin-2,4(1H,3H)-dione	7.93	902	-	65996-50-1	0.074±0.020a	0.055±0.005b	0.016±0.008c
25	(S)-(+)-5-Methyl-1-heptanol	9.98	959	-	57803-73-3	0.044±0.007a	0.036±0.006a	0.000±0.000b
26	Benzaldehyde	10.07	962	961	100-52-7	1.172±0.105b	0.797±0.232c	1.547±0.109a
27	1-Heptanol	10.56	976	969	111-70-6	0.014±0.007b	0.012±0.001b	0.033±0.028a
28	1-octen-3-ol	10.94	986	976	3391-86-4	0.169±0.009c	0.837±0.131b	1.366±0.150a
29	2-methyl-pentanoic acid anhydride	11.17	993	-	63169-61-9	0.058±0.005a	0.066±0.004a	0.024±0.002b
30	3-Octanone	11.24	995	984	106-68-3	0.039±0.034a	0.000±0.000b	0.000±0.000b
31	5-Hepten-2-one, 6-methyl-	11.27	996	985	110-93-0	0.016±0.001b	0.016±0.001b	0.025±0.003a
32	.beta.-Myrcene	11.43	1000	992	123-35-3	0.132±0.006a	0.128±0.035a	0.011±0.009b
33	3-Octanol	11.61	1005	994	589-98-0	0.012±0.000c	0.044±0.007b	0.084±0.008a
34	Decane	11.78	1010	-	124-18-5	0.011±0.001c	0.837±0.131a	0.522±0.767b
35	Hexanoic acid, ethyl ester	11.82	1011	998	123-66-0	0.016±0.001a	0.016±0.001a	0.000±0.000b
36	Octanal	11.91	1014	1005	124-13-0	0.030±0.001a	0.021±0.002a	0.004±0.000b
37	4-Hexen-1-ol, (4E)-, acetate	12.16	1021	-	1000352-71-9	0.008±0.003a	0.004±0.003b	0.005±0.006b
38	Acetic acid, hexyl ester	12.39	1027	1011	142-92-7	0.000±0.000a	0.001±0.000a	0.001±0.000a
39	1,7,7-trimethyl-bicyclo[2.2.1]hept-2-ene	12.42	1028	909	464-17-5	0.003±0.006b	0.000±0.000c	1.475±0.113a
40	o-Cymene	12.75	1037	1026	527-84-4	0.042±0.013a	0.028±0.006b	0.020±0.010b
41	D-Limonene	12.90	1041	1029	5989-27-5	0.020±0.002a	0.018±0.004a	0.004±0.001b
42	2-ethyl-1-hexanol	12.98	1044	1031	104-76-7	0.052±0.003b	0.048±0.004b	2.121±0.139a
43	1-phenyl-1,2-propanediol	13.12	1048	1326	1855-09-0	0.556±0.057b	0.566±0.057b	1.663±0.144a
44	Cyclohexanone, 2,2,6-trimethyl-	13.14	1048	1023	2408-37-9	0.012±0.000a	0.009±0.001b	0.004±0.000c
45	3,6,6-trimethyl-bicyclo[3.1.1]hept-2-ene	13.36	1054	-	4889-83-2	0.000±0.000b	0.000±0.000b	0.759±0.061a
46	Benzeneacetaldehyde	13.52	1059	1043	122-78-1	9.160±0.633a	8.502±0.755a	0.557±0.095b
47	3-Carene	13.76	1066	1018	13466-78-9	0.000±0.000b	0.000±0.000b	0.758±0.061a
48	Isophorone	14.16	1077	1118	78-59-1	0.041±0.001b	0.025±0.002c	2.423±0.252a
49	Acetophenone	14.42	1084	1068	98-86-2	0.027±0.001b	0.027±0.003b	0.278±0.023a
50	Cyclooctyl alcohol	14.57	1089	-	696-71-9	0.012±0.001a	0.014±0.001a	0.001±0.001b
51	3,3-Diethoxy-1-propyne	14.70	1092	-	10160-87-9	0.155±0.010a	0.157±0.010a	0.061±0.053b
52	Ethyl 2-(5-methyl-5-vinyltetrahydrofuran-2-yl)propan-2-yl carbonate	15.34	1110	-	1000373-80-3	0.902±0.070b	0.886±0.080b	0.963±0.100a
53	Benzene, 4-ethenyl-1,2-dimethyl-	15.37	1111	-	27831-13-6	0.006±0.002a	0.005±0.002a	0.007±0.006a
54	3,5-Octadien-2-one	15.56	1116	1098	38284-27-4	0.015±0.001b	0.014±0.002b	0.083±0.011a
55	3,7-Dimethyl-2,3,3a,4,5,6-hexahydro-1-benzofuran	15.61	1118	-	1000099-60-6	0.033±0.002c	0.062±0.048b	1.827±0.164a
56	2-Carbethoxy-N-methylpyrrolidine	15.94	1127	-	30727-23-2	0.130±0.005b	0.129±0.008b	0.192±0.018a
57	Linalool	15.95	1127	1100	78-70-6	2.247±0.115a	2.232±0.132a	0.095±0.021b
58	Nonanal	16.04	1130	1102	124-19-6	0.121±0.010a	0.107±0.006b	0.019±0.001c
59	Phenylethyl alcohol	16.29	1137	1116	60-12-8	1.785±0.142a	1.887±0.222a	0.461±0.114b

60	4-chloro-2-methyl-1-phenyl-3-buten-1-ol	15.94	1127	-	1000153-34-9	0.219±0.011b	0.217±0.013b	1.263±0.370a
61	2-Undecene, 9-methyl-, (Z)-	16.49	1143	-	74630-45-8	0.010±0.002a	0.006±0.001b	0.003±0.000c
62	3,3,5-trimethyl- heptane	15.94	1127	-	7154-80-5	0.378±0.151a	0.023±0.012b	0.016±0.004b
63	Benzene, 1-isocyano-3-methyl-	17.29	1165	-	20600-54-8	0.001±0.000c	0.005±0.001b	0.048±0.013a
64	2,4,6-Octatriene, 2,6-dimethyl-	17.41	1168	1144	673-84-7	0.020±0.003a	0.020±0.005a	0.003±0.002b
65	9-methylheptadecane	17.53	1172	-	26741-18-4	0.003±0.000b	0.005±0.001a	0.002±0.002b
66	Sulfurous acid, isobutyl pentyl ester	17.73	1178	-	1000309-13-8	0.009±0.001a	0.006±0.000b	0.005±0.002b
67	3-Ethyl-3-methylheptane	17.97	1184	-	17302-01-1	0.002±0.002b	0.001±0.000b	0.005±0.005a
68	2-Oxo-4-phenyl-6-(4-chlorophenyl)-1,2-dihydropyrimidine	18.11	1188	-	24030-13-5	0.007±0.001b	0.015±0.011a	0.004±0.000b
69	1-Nonanol	18.55	1201	1186	143-08-8	0.047±0.000b	0.061±0.002b	0.497±0.065a
70	Linalool oxide (pyranoid)	18.63	1203	1183	14049-11-7	0.192±0.017b	0.174±0.027b	7.846±0.370a
71	Naphthalene	18.86	1209	1191	91-20-3	0.033±0.002b	0.028±0.002b	0.116±0.002a
72	2-methoxy-3-(2-methylpropyl)-Pyrazine	18.89	1210	1187	24683-00-9	0.003±0.000b	0.003±0.000b	0.615±0.022a
73	(Z)-butanoic acid, 3-hexenyl ester	19.14	1217	1186	16491-36-4	0.022±0.004c	0.035±0.006b	1.317±1.003a
74	L-α-Terpineol	19.23	1220	1187	10482-56-1	0.008±0.007b	0.014±0.003b	0.121±0.008a
75	1-dodecanol	19.30	1222	1188	112-53-8	0.001±0.001b	0.001±0.001b	2.032±0.133a
76	Methyl salicylate	19.36	1223	1190	119-36-8	0.722±0.179a	0.690±0.059a	0.374±0.566b
77	1,3-Cyclohexadiene-1-carboxaldehyde, 2,6,6-trimethyl-	19.37	1224	1197	116-26-7	0.029±0.012b	0.027±0.002b	0.036±0.007a
78	Dodecane	19.62	1231	-	112-40-3	0.028±0.002b	0.032±0.007b	0.068±0.008a
79	Decanal	19.81	1236	1200	112-31-2	0.009±0.001a	0.011±0.001a	0.005±0.000b
80	Decane, 5-ethyl-5-methyl-	20.04	1243	-	17312-74-2	0.008±0.001b	0.011±0.001a	0.000±0.000c
81	1-Cyclohexene-1-carboxaldehyde, 2,6,6-trimethyl-	20.33	1251	1214	432-25-7	0.084±0.003a	0.059±0.004b	0.068±0.002b
82	cis-3-Hexenyl-α-methylbutyrate	20.80	1264	1233	53398-85-9	0.061±0.003b	0.067±0.005b	1.124±0.323a
83	n-Valeric acid cis-3-hexenyl ester	20.93	1268	1235	35852-46-1	0.017±0.001b	0.021±0.003b	15.670±4.899a
84	Valeric anhydride	20.95	1268	-	2082-59-9	0.010±0.001a	0.009±0.001a	0.000±0.000b
85	Formic acid dodecyl ester	21.07	1272	-	28303-42-6	0.026±0.001b	0.026±0.002b	1.081±0.098a
86	Nerol	21.57	1286	1229	106-25-2	1.410±0.044a	1.425±0.136a	0.170±0.070b
87	6-methyl-pentadecane	21.95	1296	-	10105-38-1	0.004±0.003b	0.002±0.004b	2.638±1.95a
88	2-hydroxy-benzoic acid ethyl ester	22.11	1301	1267	118-61-6	0.009±0.002b	0.008±0.001b	0.386±0.033a
89	m-Aminophenylacetylene	22.86	1322	-	54060-30-9	0.005±0.001b	0.004±0.001b	0.046±0.002a
90	Tridecane	23.15	1330	-	629-50-5	0.013±0.002b	0.012±0.001b	0.035±0.008a
91	7-Tetradecene	23.87	1350	-	10374-74-0	0.002±0.000b	0.003±0.000b	0.007±0.001a
92	Dodecane, 1-iodo-	24.03	1355	-	4292-19-7	0.042±0.006a	0.041±0.001a	0.021±0.004b
93	Heptadecane, 7-methyl-	24.35	1364	-	20959-33-5	0.023±0.003a	0.027±0.002a	0.025±0.002a
94	Nonadecane, 9-methyl-	24.47	1367	-	13287-24-6	0.001±0.000b	0.002±0.000b	0.005±0.000a
95	(E)-Hex-3-enyl (E)-2-methylbut-2-enoate	24.53	1369	-	1000373-74-1	0.005±0.001b	0.007±0.001ab	0.009±0.002a
96	Eicosane, 10-methyl-	24.71	1374	-	54833-23-7	0.011±0.001a	0.013±0.001a	0.011±0.007a
97	Tridecane, 5-methyl-	24.94	1381	-	25117-31-1	0.006±0.001b	0.007±0.001b	0.015±0.001a
98	Tetradecane, 4-methyl-	25.49	1396	1377	25117-24-2	0.013±0.002c	0.027±0.003b	0.068±0.007a
99	(Z)-hexanoic acid, 3-hexenyl ester	25.88	1407	1381	31501-11-8	0.175±0.003b	0.224±0.014a	0.035±0.006c
100	cis-3-Hexenyl cis-3-hexenoate	26.04	1412	1389	61444-38-0	0.063±0.094a	0.012±0.001b	0.010±0.001b
101	1-Tetradecanol	26.20	1416	1396	112-72-1	0.006±0.000c	0.011±0.003b	0.022±0.003a
102	2-Cyclopenten-1-one, 3-methyl-2-(2-pentenyl)-, (Z)-	26.43	1422	1402	488-10-8	0.011±0.000a	0.011±0.003a	0.002±0.000b
103	Tetradecane	26.46	1423	-	629-59-4	0.045±0.002c	0.063±0.004b	0.156±0.020a
104	2-epi-α-Funebrene	26.88	1435	1419	65354-33-8	0.003±0.000b	0.004±0.000b	0.007±0.001a
105	α-ionone	27.36	1449	1426	127-41-3	0.029±0.001b	0.027±0.003b	0.728±0.054a
106	Pentadecafluorooctanoic acid, tetradecyl ester	27.96	1466	-	1000406-04-4	0.004±0.001b	0.006±0.000b	0.012±0.002a
107	5,9-Undecadien-2-one, 6,10-dimethyl-, (E)-	28.14	1471	1458	3796-70-1	0.015±0.000b	0.012±0.001b	0.097±0.008a
108	2,6,10-Trimethyltridecane	28.41	1478	1461	3891-99-4	0.005±0.001b	0.008±0.001b	0.026±0.006a
109	Tetradecane, 3-methyl-	28.65	1485	1468	18435-22-8	0.004±0.003b	0.008±0.001a	0.002±0.002b
110	*trans*-β-ionone	29.18	1500	1486	79-77-6	0.420±0.003b	0.284±0.040c	1.242±0.095a
111	n-Pentadecanol	29.33	1504	1492	629-76-5	0.003±0.000b	0.004±0.001b	0.012±0.003a
112	Pentadecane	29.57	1511	-	629-62-9	0.011±0.001b	0.017±0.001b	0.035±0.007a
113	Sulfurous acid, butyl tetradecyl ester	29.74	1516	-	1000309-18-1	0.009±0.002a	0.009±0.000a	0.007±0.003a
114	Pentanoic acid, 5-hydroxy-, 2,4-di-t-butylphenyl esters	29.94	1521	-	166273-38-7	0.045±0.002a	0.045±0.008a	0.034±0.007b
115	Butylated Hydroxytoluene	30.00	1523	1511	128-37-0	0.035±0.002b	0.041±0.003b	0.109±0.009a
116	Naphthalene, 1,2,3,4-tetrahydro-1,6-dimethyl-4-(1-methylethyl)-, (1S-cis)-	30.33	1532	1517	483-77-2	0.060±0.002a	0.038±0.010b	0.013±0.007c
117	6-Tetradecanesulfonic acid, butyl ester	30.45	1536	-	1000280-27-4	0.004±0.000c	0.009±0.001b	0.017±0.004a
118	Undecane, 3,8-dimethyl-	30.83	1546	-	17301-30-3	0.023±0.004b	0.021±0.001b	0.033±0.006a

119	1,2-Benzenediol, O-(1-naphthoyl)-	31.03	1552	-	1000325-94-2	0.054±0.006b	0.075±0.004a	0.011±0.001c
120	n-Nonylcyclohexane	31.13	1555	1556	2883-02-5	0.006±0.001b	0.010±0.001a	0.008±0.013ab
121	1,6,10-Dodecatrien-3-ol, 3,7,11-trimethyl-, (E)-	31.63	1569	1569	40716-66-3	0.058±0.002a	0.050±0.007a	0.005±0.001b
122	Pentadecane, 3-methyl-	31.85	1575	1570	2882-96-4	0.005±0.000c	0.009±0.001b	0.019±0.003a
123	3-Hexen-1-ol, benzoate, (Z)-	31.88	1576	1573	25152-85-6	0.019±0.002a	0.015±0.000a	0.019±0.020a
124	Glutaric acid, butyl isobutyl ester	32.17	1584	-	1000358-25-1	0.021±0.002a	0.022±0.000a	0.018±0.002a
125	Cyclopropane, 1-methyl-1-(1-methylethyl)-2-nonyl-	32.40	1591	-	41977-40-6	0.004±0.000c	0.009±0.000b	0.016±0.015a
126	1-Hexadecanol	32.61	1597	1609	36653-82-4	0.003±0.000b	0.004±0.000b	0.007±0.002a
127	Hexadecane	32.84	1603	-	544-76-3	0.028±0.002b	0.033±0.002b	0.074±0.019a
128	5,5-Diethyltridecane	32.92	1605	-	1000360-41-3	0.001±0.000a	0.001±0.000a	0.002±0.002a
129	Adipic acid, butyl isobutyl ester	34.97	1663	-	1000324-09-3	0.009±0.001a	0.009±0.001a	0.010±0.002a
130	Heptadecane	35.24	1671	-	629-78-7	0.008±0.001b	0.008±0.001b	0.012±0.002a
131	Pentadecane, 2,6,10,14-tetramethyl-	35.36	1674	1707	1921-70-6	0.006±0.001a	0.008±0.001a	0.007±0.002a
132	Undecane, 4,4-dimethyl-	36.18	1702	-	17312-68-4	0.003±0.000b	0.004±0.001b	0.007±0.002a
133	Heptadecane, 3-methyl-	36.51	1735	1771	6418-44-6	0.002±0.000b	0.002±0.000b	0.004±0.001a
134	Decane, 3,8-dimethyl-	36.96	1780	1804	17312-55-9	0.007±0.002b	0.008±0.003b	0.031±0.033a
135	2-Ethylhexyl salicylate	37.09	1792	1816	118-60-5	0.005±0.002c	0.010±0.004b	0.037±0.038a
136	Caffeine	37.65	1848	1842	58-08-2	0.128±0.012a	0.119±0.028a	0.051±0.051b
137	Phthalic acid, hept-4-yl isobutyl ester	37.98	1881	-	1000356-78-3	0.115±0.016a	0.089±0.020b	0.070±0.025c
138	Borane, diethyl(decyloxy)-	38.34	1916	-	1000152-34-3	0.001±0.000b	0.001±0.000b	0.006±0.002a
139	Dibutyl phthalate	39.16	1997	1967	84-74-2	0.065±0.015b	0.052±0.008c	0.096±0.041a
140	Hexadecanoic acid, ethyl ester	39.48	2006	1978	628-97-7	0.069±0.006a	0.041±0.009b	0.067±0.056a
141	Neophytadiene	40.75	2155	1840	504-96-1	0.024±0.002a	0.014±0.002b	0.016±0.004b

Note: RI[a]: the linear retention indices calculated from a series of n-alkanes (C7-C40); RI[b]: retention indices referred to the literature value with HP-5MS column or equivalent chromatographic column [NIST Chemistry WebBook (http://webbook.nist.gov/chemistry]. Data are presented as mean ± standard deviation (n=3). Mean values with different lowercase letters in the same column indicate significant differences based on the least significant difference (LSD) test ($p < 0.05$).

Table S2. The contents and proportion of different volatile metabolites types in BT, BoS, and STL.

Volatiles types		BT	BoS	STL
Alcohols	Content (μg/g)	8.333±0.425c	9.678±0.410b	17.947±0.607a
	proportion	0.301±0.003b	0.360±0.008a	0.317±0.026b
Esters	Content (μg/g)	2.375±0.213b	2.288±0.193b	21.343±5.100a
	proportion	0.086±0.009b	0.085±0.005b	0.372±0.051a
Aldehydes	Content (μg/g)	12.827±0.779a	11.097±0.744b	2.479±0.218c
	proportion	0.463±0.008a	0.412±0.014b	0.044±0.003c
Ketones	Content (μg/g)	0.639±0.042b	0.439±0.045c	4.885±0.366a
	proportion	0.023±0.002b	0.016±0.001c	0.086±0.008a
Enynes and Alkynes	Content (μg/g)	0.437±0.031b	0.397±0.057b	3.206±0.193a
	proportion	0.016±0.001b	0.015±0.002b	0.057±0.004a
Aromatic hydrocarbons	Content (μg/g)	1.765±0.063b	1.224±0.179c	2.912±0.328a
	proportion	0.064±0.006a	0.046±0.008b	0.051±0.003b
Alkanes	Content (μg/g)	0.927±0.553c	1.450±0.195b	4.069±1.271a
	proportion	0.033±0.019c	0.054±0.005b	0.071±0.021a
Others	Content (μg/g)	0.367±0.014a	0.348±0.067a	0.107±0.018b
	proportion	0.013±0.001a	0.013±0.003a	0.002±0.000b

Total content of aroma substances（μg/g）	27.669±1.335b	26.921±1.284b	56.948±6.912a

Note: BT, black tea; BoS, black tea and *Strobilanthes tonkinensis* Lindau; STL, *Strobilanthes tonkinensis* Lindau. Data are presented as mean ± standard deviation (n=3). Mean values with different lowercase letters in the same column indicate significant differences based on the least significant difference (LSD) test ($p < 0.05$).

Table S3. List of the differential volatile metabolites among BT, BoS, and STL.

No.	Name	BT	BoS	STL
13	Hexanal	0.396±0.020a	0.223±0.084b	0.042±0.007c
14	(E)-2-hexenal	1.214±0.039a	0.661±0.117b	0.042±0.008c
15	1-Methyl-1H-1,2,4-triazole	1.214±0.039a	0.661±0.117b	0.042±0.008c
16	(E)-3-hexen-1-ol	0.774±0.062b	0.963±0.229a	0.058±0.011c
18	Cis-2-methyl-cyclopentanol	0.085±0.006b	0.176±0.036a	0.011±0.015c
19	1-hexanol	0.489±0.019b	0.751±0.191a	0.020±0.005c
26	Benzaldehyde	1.172±0.105b	0.797±0.232c	1.547±0.109a
28	1-octen-3-ol	0.169±0.009c	0.837±0.131b	1.366±0.150a
34	Decane	0.011±0.001c	0.837±0.131a	0.522±0.767b
39	1,7,7-trimethyl-bicyclo[2.2.1]hept-2-ene	0.003±0.006b	0.000±0.000c	1.475±0.113a
42	2-ethyl-1-hexanol	0.052±0.003b	0.048±0.004b	2.121±0.139a
43	1-phenyl-1,2-propanediol	0.556±0.057b	0.566±0.057b	1.663±0.144a
45	3,6,6-trimethyl-bicyclo[3.1.1]hept-2-ene	0.000±0.000b	0.000±0.000b	0.759±0.061a
46	Benzeneacetaldehyde	9.160±0.633a	8.502±0.755a	0.557±0.095b
47	3-Carene	0.000±0.000b	0.000±0.000b	0.758±0.061a
48	Isophorone	0.041±0.001b	0.025±0.002c	2.423±0.252a
55	3,7-Dimethyl-2,3,3a,4,5,6-hexahydro-1-benzofuran	0.033±0.002c	0.062±0.048b	1.827±0.164a
57	Linalool	2.247±0.115a	2.232±0.132a	0.095±0.021b
59	Phenylethyl alcohol	1.785±0.142a	1.887±0.222a	0.461±0.114b
60	4-chloro-2-methyl-1-phenyl-3-buten-1-ol	0.219±0.011b	0.217±0.013b	1.263±0.370a
62	3,3,5-trimethyl-heptane	0.378±0.151a	0.023±0.012b	0.016±0.004b
70	Linalool oxide (pyranoid)	0.192±0.017b	0.174±0.027b	7.846±0.370a
72	2-methoxy-3-(2-methylpropyl)-Pyrazine	0.003±0.000b	0.003±0.000b	0.615±0.022a
73	(Z)-butanoic acid, 3-hexenyl ester	0.022±0.004c	0.035±0.006b	1.317±1.003a
75	1-dodecanol	0.001±0.001b	0.001±0.001b	2.032±0.133a
82	cis-3-Hexenyl-α-methylbutyrate	0.061±0.003b	0.067±0.005b	1.124±0.323a
83	n-Valeric acid cis-3-hexenyl ester	0.017±0.001b	0.021±0.003b	15.670±4.899a
85	Formic acid dodecyl ester	0.026±0.001b	0.026±0.002b	1.081±0.098a
86	Nerol	1.410±0.044a	1.425±0.136a	0.170±0.070b
87	6-methyl-pentadecane	0.004±0.003b	0.002±0.004b	2.638±1.95a
99	(Z)-hexanoic acid, 3-hexenyl ester	0.175±0.003b	0.224±0.014a	0.035±0.006c
105	α-ionone	0.029±0.001b	0.027±0.003b	0.728±0.054a
110	*trans*-β-ionone	0.420±0.003b	0.284±0.040c	1.242±0.095a

Note: Data are presented as mean ± standard deviation (n=3). Mean values with different lowercase letters in the same column indicate significant differences based on the least significant difference (LSD) test ($p < 0.05$).
